# 

**Published:** 1876-12

**Authors:** 


### CONTENTS:

ORIGINAL COMMUNICATIONS:

Therapeutics of Epilepsy. By A. McLane Hamilton, M D. .......... 1057

Experimental Study of the Process of Repair. By I. N. Danforth, M. D. .......... 1071

Subperitoneal Fibroid Tumor of the Uterus Removed through an Incision in the Posterior Wall of the Vagina. By R. Stansbury Sutton, M.D. .......... 1079

Mortality of Surgical Operations in the Upper Lake States, compared with that of other Regions. By E. Andrews, M. D. .......... 1087

Case of Stomatitis Materna and Intricate Diarrhœa complicating each Gestation and Lactation; with Dropsy of the Amnion, and premature Expulsion of an Emphysematous Living Fœtus in the last Pregnancy. By J. Schneck, M. D. .......... 1103

EDITORIAL .......... 1107

HOSPITALS .......... 1108

BOOK REVIEWS .......... 1116

BOOKS AND PAMPHLETS RECEIVED .......... 1125

EXCHANGES .......... 1127

SUMMARY OF PROGRESS IN THE MEDICAL SCIENCES:

I. Therapeutics .......... 1129

II. Practical Medicine .......... 1134

III. Surgery .......... 1140

ANNOUNCEMENTS FOR THE MONTH .......... 1144

